# Grading operative findings at laparoscopic cholecystectomy- a new scoring system

**DOI:** 10.1186/s13017-015-0005-x

**Published:** 2015-03-08

**Authors:** Michael Sugrue, Shaheel M Sahebally, Luca Ansaloni, Martin D Zielinski

**Affiliations:** Department of Surgery, Letterkenny Hospital and Donegal Clinical Research Academy, National University Ireland Galway, Letterkenny, Donegal Ireland; Department of Surgery, Papa Giovanni XXIII Hospital, Bergamo, Italy; Department of Surgery, Mayo Clinic, Rochester, Minnesota, USA

**Keywords:** Cholecystitis, Cholecystectomy, Laparoscopic, Operative severity scoring system, Conversion to open

## Abstract

**Introduction:**

Variation in outcomes from surgery is a major challenge and defining surgical findings may help set benchmarks, which currently do not exist in laparoscopic cholecystectomy. This study outlines a new surgical scoring system incorporating key operative findings.

**Methods:**

English language studies (from January 1965 to July 2014) pertaining to severity scoring and predictors of difficult laparoscopic cholecystectomy were searched for in PubMed, Embase and Cochrane databases using the search terms ‘Laparoscopic cholecystectomy or Lap chole’ and/or ‘Scoring Index or Grading system or Prediction of difficulty or Conversion to open’ in various combinations. Cross-referencing from papers retrieved in the original search identified additional articles.

**Results:**

Sixteen published papers report a gallbladder (GB) scoring system, but all relate to pre-operative clinical and imaging findings, rather than operative findings. The current scoring system, using operative findings incorporates the appearance of the GB, presence of GB distension, ease of access, potential biliary complications and time taken to identify cystic duct and artery. A score of <2 would imply mild difficulty, 2–4 moderate, 5–7 severe and 8–10 extreme.

**Conclusion:**

This paper reports one of the first operative classifications of findings at laparoscopic cholecystectomy. It has the potential to allow benchmarks for international collaboration of operative and patient outcomes in patients undergoing laparoscopic cholecystectomy.

## Introduction

Gallbladder-related disease is now one of the commonest indications for elective and emergency surgery. Management of cholecystitis and its complications has evolved dramatically [1] and there have been significant paradigm shifts in the management of patients since the introduction of laparoscopic cholecystectomy in the mid 1990 [2]. Recently the importance of index admission laparoscopic cholecystectomy has been highlighted [3]. In many large series and meta-analyses detailed patient demographics and imaging findings have been recorded. A number of international guidelines recommend pathways of care [4,5]. Attempts have been made to standardize definitions particularly relating to cholecystitis [6]. Understanding outcomes is key to advancing health care, and while conversion to open cholecystectomy will always be an essential part of safe surgical practice, a greater understanding of the factors leading to conversion and potential post-operative complications would be essential.

Despite these advances, significant variability in approaches to care and outcomes in gall-bladder disease management are reported [7]. While a number of pre-operative scoring systems are reported there is no operative classification of findings at laparoscopic surgery [8,9]. This limits the ability to compare outcomes or provide a common benchmark for future research. This paper outlines a new scoring system for operative findings at laparoscopic cholecystectomy, to allow grading of the findings and standardize the degree of cholecystitis.

## Methods

A literature review was undertaken of PubMed, Embase and Cochrane databases between January 1965 and July 2014 for publications relating to difficulty prediction in laparoscopic cholecystectomy using the search terms ‘Laparoscopic cholecystectomy or Lap chole’ and/or ‘Scoring Index or Grading system or Prediction of difficulty or Conversion to open’ in various combinations. Cross-referencing from papers retrieved in the original search identified additional articles. All studies had to be published in English literature. Case reports and data from abstracts were excluded.

## Results

In total 16 papers were found relating to difficulty prediction in laparoscopic cholecystectomy. These are summarised in Table 1. All papers focused on the ability to predict conversion to open surgery using preoperative parameters. No operative grading system was found. Our study provides a preliminary scoring system to enable key aspects of the surgical findings to be documented.

**Table 1:** **Summary of studies reporting severity scoring system for laparoscopic cholecystectomy**

**Study details**	**Statistically significant clinical parameters**	**Statistically significant radiological parameters**	**Statistically significant intra-operative parameters**	**Comments**
Vivek et al. Prospective (n = 323)	Male gender, Previous attacks of AC, Previous upper abdominal surgery	Multiple stones Peripancreatic fluid collection	Cirrhotic liver Contracted/distended GB Inflamed GB Ductal anomalies Adhesions	Max score of 44 (with 9 predicting difficult LC), sensitivity of 85% & specificity of 97.8%. ROC of 0.96.
Gupta et al. Prospective (n = 210) All underwent elective LC.	History of previous hospitalization due to AC, Palpable GB	Thickened (≥4 mm) GB wall, Impacted stone	N/A	Min score 0 (easy) Max score 15 (very difficult). Conversion rate 4.28% ROC of 0.86. PPV for easy and difficult LC were 90% and 88% respectively.
Randhawa et al. Prospective (n = 228)	BMI >27.5, Previous hospitalization due to AC, Palpable GB	Thickened (≥4 mm) GB wall	N/A	Conversion rate of 1.31%. ROC of 0.82. PPV for easy and difficult LC were 88.8% and 92.2% respectively.
Kanakala et al. Initially retrospective then prospective (n = 2117)	Male gender, ASA II and III	N/A	N/A	Conversion rate of 6.3%.
Bouarfa et al. Retrospective (n = 337) All underwent elective LC.	Male gender, High BMI	GB wall thickening (>2 mm), GB wall inflammation	N/A	Classification algorithms based on preoperative patient data to predict intraoperative complexity, with an accuracy of 83%.
Kama et al. Retrospective (n = 1000)	Age ≥ 60 (p = 0.052), Male gender, Abdominal tenderness, Previous upper abdominal operation	Thickened GB wall (>4 mm), Previous attacks of AC	N/A	Conversion rate of 4.8%. Both a constant and coefficient were calculated for each parameter; the sum of both gives a score for the patient
Kologlu et al. Prospective (n = 400)				This was a validation of the study by Kama et al. using the RSCLO score. Increasing RSCLO scores correlated with higher conversion rates. Conversion rate of 3%.
Lal P et al. Prospective (n = 73) All underwent elective LC.	N/A	GB wall thickness (>4 mm), Contracted GB, Stone impaction at Hartmann’s pouch.	Total operating time (>90mins), Time taken to dissect GB bed/Calot’s triangle (>20 mins), Spillage of stones, Tear of GB during dissection, Conversion to open were chosen as parameters describing a difficult LC.	Conversion rate of 23.3%. PPV of GB thickness, stone impaction and contracted GB to predict conversion to open were 70%, 63.6% and 45.4%, respectively, with a combined overall ultrasonographic PPV of 61.9%.
Schrenk et al. Prospective with 2 arms (n = 640 altogether)	RUQ pain, Rigidity in RUQ, Previous upper abdominal surgery, biliary colic in last 3 weeks, WCC > 10 x 10^9^/L	GB wall thickening (>5 mm), Hydroptic GB, Pericholecystic fluid, Shrunken GB, No GB filling on preoperative IV cholangiography/incarcerated cystic duct stone (on U/S)	N/A	Conversion rate of 8.2%. 5 possible scores, ranging from 0–9 (with 0 = easy LC and ≥4 = conversion to open expected). PPV of 80%.
Rosen et al. Retrospective (n = 1347) undergoing both elective and non-elective LC.	Age, BMI, AC	GB wall thickness	N/A	Conversion rate of 5.3%. For elective LC, BMI >40 and GB wall thickness > 4 mm predicted conversion. For non-elective LC, ASA >2 predicted conversion.
Nachnani et al. Prospective (n = 105)	Male gender, Previous abdominal surgery, BMI > 30, Previous AC/acute pancreatitis	GB wall thickness > 3 mm	N/A	Conversion rate of 11.4%.
Abdel-Baki et al. (n = 40)	N/A	GB wall thickness (≥3 mm), Liver fibrosis	N/A	Conversion rate of 0.42%.
Daradkeh et al. Prospective (n = 160)	N/A	GB wall thickness (>3 mm), CBD diameter (≥7 mm)	N/A	Conversion rate of 2.5%. Adjusted *r* ^2^ for U/S parameters was 0.25.
Bulbuller et al. Prospective (n = 571)	N/A	N/A	N/A	Conversion rate of 3.3%. Evaluation of RSCLO score showed good correlation with conversion to open, with a PPV of 43%, NPV of 100%, sensitivity of 100% and specificity of 96%.
Kwon et al. Retrospective (n = 305) All patients underwent ERCP and EST prior to LC (acute or elective).	See comments	See comments	See comments	This study evaluated risk factors for conversion to open surgery in patients who underwent prior ERCP and EST for choledochocystolithiasis. Cholecystitis, mechanical lithotripsy and ≥ 2 CBD stones predicted open surgery. Conversion rate of 15.7%.
Lipman et al. Retrospective (n = 1377)	Male gender, Elevated WCC (≥11,000/μL), Low serum albumin (<3.5 g/dL), Diabetes Mellitus, Elevated total bilirubin (≥1.5 g/dL)	Pericholecystic fluid	N/A	Conversion rate of 8.1%. ROC of model was 0.83.

AC: acute cholecystitis; LC: laparoscopic cholecystectomy; GB: gallbladder; ASA: American Society of Anaesthesiologists; BMI: body mass index; RUQ: right upper quadrant; WCC: white cell count; ERCP: endoscopic retrograde cholangiopancreatography; EST: endoscopic sphincterotomy.

The current scoring system proposed is based on the severity of cholecystitis and degree of potential difficulty with a score from 1 to 10. The key aspects of the score include access to the gallbladder including patient body mass index (BMI), the degree of pericholic and right upper quadrant adhesions particularly in patients who have had previous abdominal surgery, the presence of complicated cholecystitis and the time taken by the surgeon to achieve the triangle of safety [10] with identification of the cystic artery and duct. With this scoring system a score of <2 would be considered easy, 2 to 4 moderate, 5–7 very difficult, and 8 to 10, extreme.

**Figure 1:**
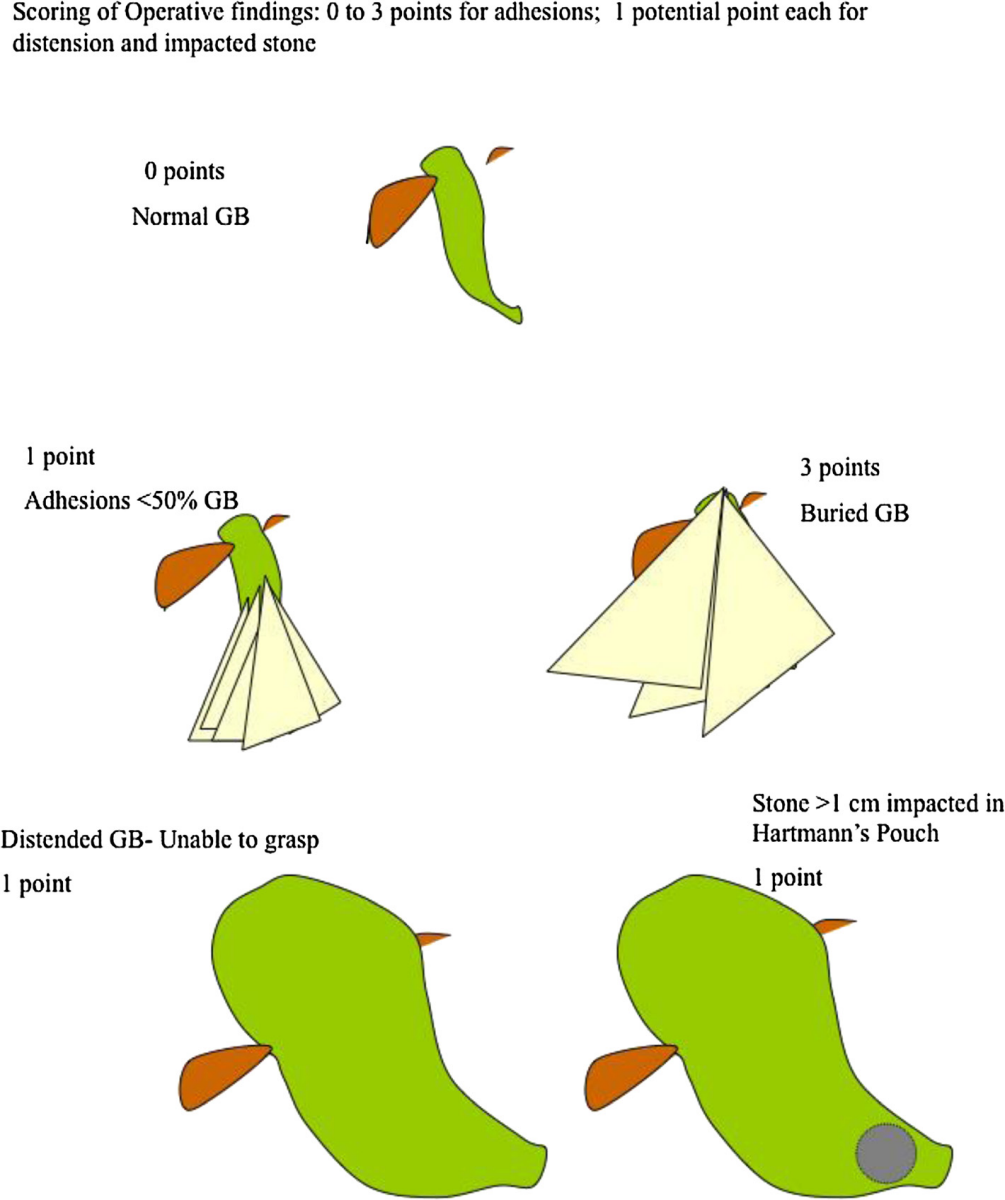
**Schematic illustration of various intraoperative findings, with their respective scores: a.** Normal gallbladder with no adhesions: 0 points. **b.** Adhesions covering < 50% of gallbladder: 1 point. **c.** Adhesions completely burying gallbladder: 3 points. **d.** Distended gallbladder, with inability to grasp with atraumatic laparoscopic forceps: 1 point. **e.** Large (>1 cm) stone impacted in Hartmann’s pouch: 1 point.

**Figure 2:**
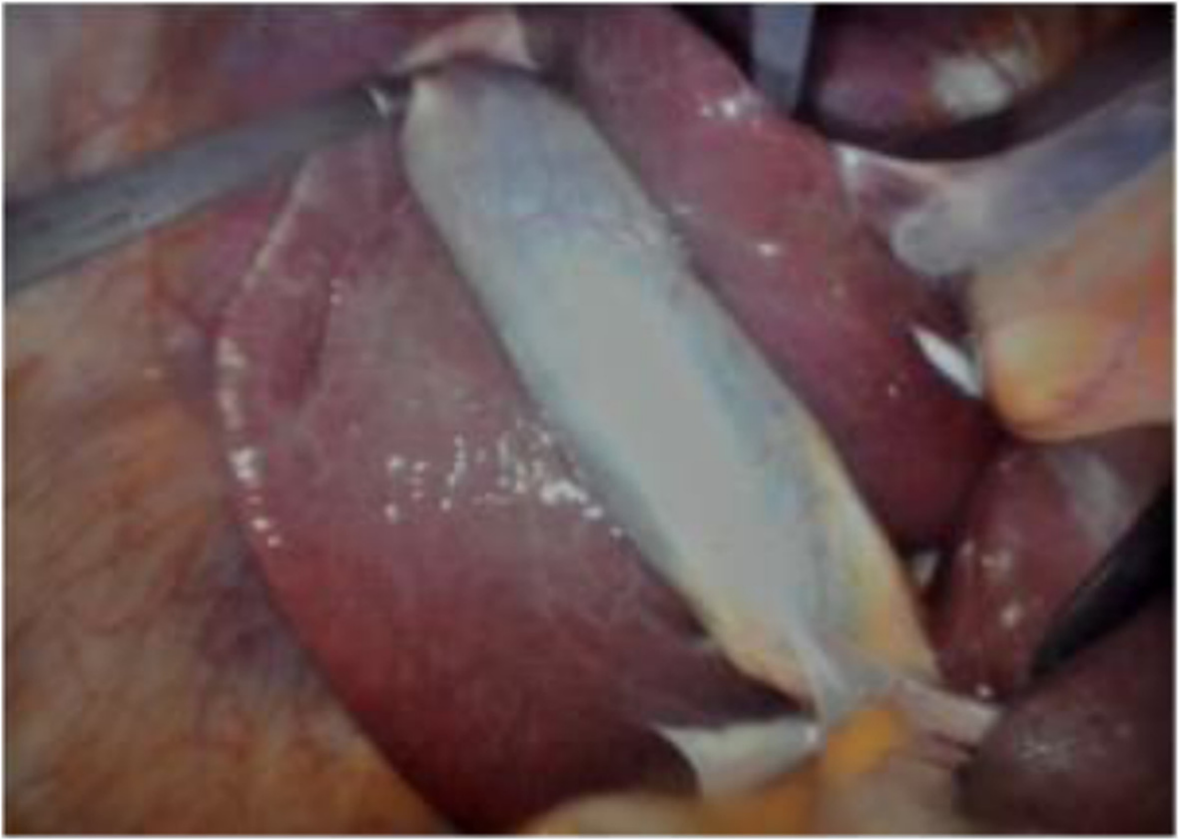
**Intraoperative image demonstrating < 50% of gallbladder covered by adhesions (1 point).**

**Figure 3:**
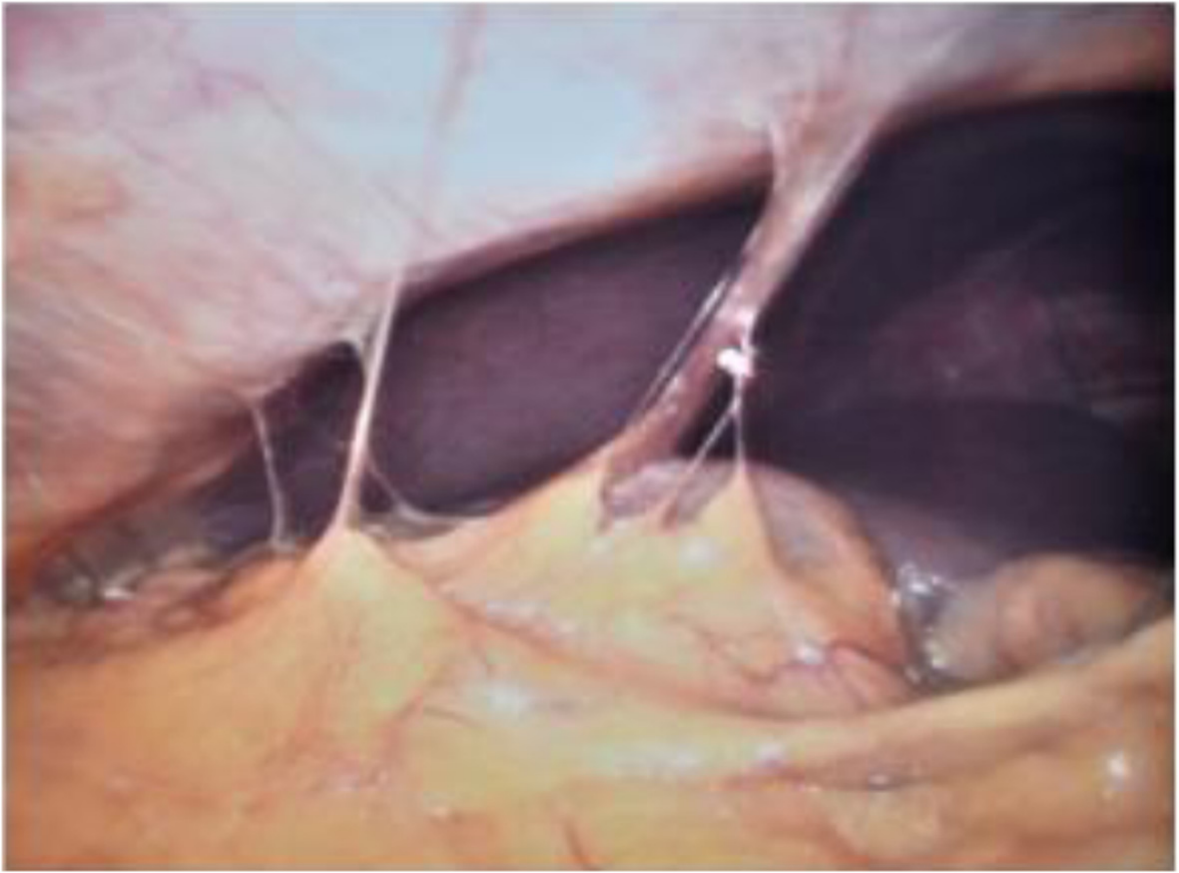
**Intraoperative image demonstrating gallbladder completely buried in adhesions (3 points).**

**Figure 4:**
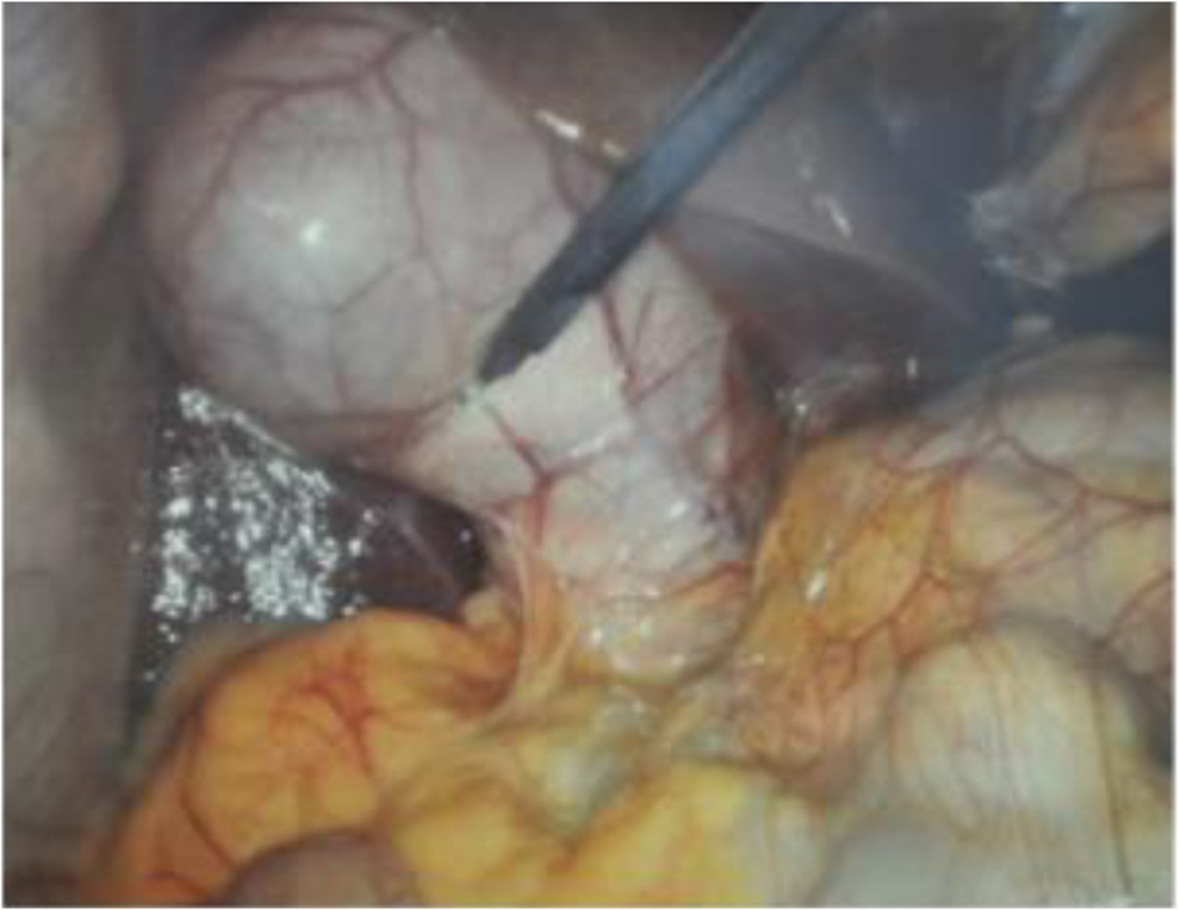
**Intraoperative image demonstrating a distended gallbladder (1 point), with < 50% of its surface area covered by adhesions (1 point).**

**Figure 5:**
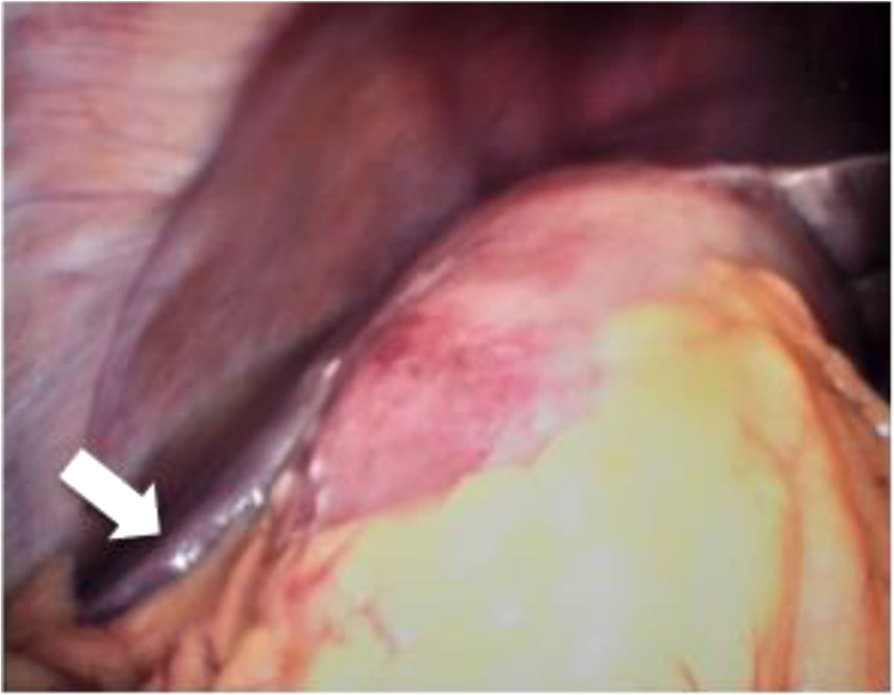
**Intraoperative image demonstrating severe sepsis/complications, with free bile (1 point, arrow) outside a distended (I point) gallbladder, covered by adhesions (3 points).**

Fistulation of the gallbladder which would be associated with extreme difficulty and a high rate of conversion was not included in the score, given its rarity and potential to skew a simple scoring system. The five key aspects include: 1) gallbladder appearance and amount of adhesions, 2) degree of distension/contracture of the gallbladder, 3) ease of access, 4) local/septic complications, and, 5) time taken to identify the cystic artery and duct (Table 2). Where there are no adhesions, a score of zero is given. The maximum achievable score for adhesions is 3, which would occur if the gallbladder were completely buried in adhesions. A distended gallbladder receives a score of 1. Failure to grasp the gallbladder with a standard, atraumatic laparoscopic forceps scores a further point. This applies either with or without adhesions present. If decompression is performed to allow grasping then a point is still awarded. Further points are awarded for access difficulties (i.e. port placement difficulties using Hasson’s technique) and complicated cholecystitis with perforation. The different grades and points are shown in Figures 1, 2, 3, 4, 5. The patient in Figure 5 would get a total of 7 points: 3 for adhesions, 1 for distended gallbladder, 1 for obesity, 1 for free fluid and 1 for a large (>1 cm) stone impacted in Hartmann’s pouch. If you could not grasp the gallbladder with a standard forceps a further point would be given.

**Table 2:** **Operative Grading System for Cholecystitis Severity**

**Gallbladder appearance**							
Adhesions < 50% of GB					1		
Adhesions burying GB					3		
				*Max*	*3*		
**Distension/Contraction**							
Distended GB (or contracted shrivelled GB)					1		
Unable to grasp with atraumatic laparoscopic forceps					1		
Stone ≥1 cm impacted in Hartman’s Pouch					1		
**Access**							
BMI >30					1		
Adhesions from previous surgery limiting access					1		
**Severe Sepsis/Complications**							
Bile or Pus outside GB					1		
**Time to identify cystic artery and duct >90 minutes**					1		
				Total *Max*	*10*		
Degree of difficulty							
A Mild	<2						
B Moderate	2–4						
C Severe	5–7						
D Extreme	8–10

## Discussion

Cholecystectomy is currently one of the commonest reasons for admission to hospital with an associated mortality of 0.45 to 6% depending on severity of gallbladder disease [11]. It accounts for a significant workflow in gastrointestinal surgery and emergency care [4,12]. Optimising care and care pathways requires an understanding of the underlying disease [13,14]. Not only can the natural history of gallbladder disease vary with patient cohorts but surgical findings can be surprising, with somewhat unexpected degrees of surgical difficulty (or ease) [15]. It is one of the more unpredictable operations in general surgery, due to the variable operative findings. Publications reporting outcomes, including conversion to open surgery, are hard to compare as currently there is no grading or scoring of operative findings at surgery [16,17].

There are some well-reported models of grading and classification systems that have laid the foundation for collaborative research and improved outcomes [18,19]. The importance of disease classification is increasingly recognised. Crandall and colleagues [20] provide a grading system for measuring anatomic severity of several Emergency General Surgery (EGS) diseases based on the American Association for the Surgery of Trauma (AAST) uniform grading system. Grading and scoring surgical conditions provide a uniform tool for reporting disease severity. As many have only been recently developed, they need validation as does the current scoring system.

The aetiology underlying variable outcomes from laparoscopic cholecystectomy is complex in origin, relating to disease severity, surgical experience, and available instrumentation. Laparoscopic cholecystectomy is now the gold standard replacing open cholecystectomy. It is accepted that recovery is delayed, and risk of complications compounded by both delayed emergency cholecystectomy and excessive conversion from laparoscopic to open surgery. Account needs to be taken, however, that a specialist hepatobiliary surgeon may have a lower conversion rate than general surgeons. However, comparisons between surgeons, institutions and published series are currently impossible as the denominator of the severity of cholecystitis is not only not standardized but also rarely reported.

Lal [15] and colleagues suggest that a difficult cholecystectomy is one taking longer that 90 minutes, tearing the gallbladder, spending more that 20 minutes dissecting the gallbladder adhesions, or more than 20 minutes dissecting Calot’s triangle. While time to dissection of Calot’s triangle will vary on surgical skills and level of experience, it will generally be longer in patients with increasing access difficulty, inflammation and adhesions. Predicting a difficult cholecystectomy is possible with some degree of accuracy, using patient demographics, BMI, presence of a palpable gallbladder, and pre-operative ultrasound (US) or computed tomography (CT) findings [8,9]. In addition, previous cholecystitis or lithotripsy has been shown to increase the likelihood of a difficult procedure [21].

With increasing pressure to perform acute index admission laparoscopic cholecystectomy, an intraoperative-based scoring system will potentially allow meaningful comparison of outcomes [22]. In addition it may provide a trigger to prompt earlier conversion or link specific outcomes measures such as bile leaks to specific operative scores.

However, the current scoring system has some limitations. It has not been validated in a large series and has some subjectivity in terms of the percentage of the gallbladder covered by adhesion. Also, it is difficult to objectively define the amount of adhesions from previous abdominal surgery. In addition adhesions may vary in tenacity and vascularity. However these are difficult to define objectively and as such have omitted from the scoring system. It is, however, simple to calculate and provides a score out of ten. Another limitation is that it does not particularly take into account intra-operative bleeding. The actual amount of bleeding is hard to measure objectively outside a clinical trial.

Other international scoring systems have facilitated advances in clinical and research into different areas of surgery [18-20,23]. Some scoring systems, like some of the previously published gallbladder related reports, have focused on prediction of outcomes from clinical and preoperative investigations rather than operative findings.

Vivek *et al*. [9] recently reported scoring assessment of difficulty in over 300 patients undergoing laparoscopic cholecystectomy and were accurate in predicting the difficulty and need for conversion. Vivek’s grading system, however, is complex using 22 parameters including 4 intra-operative parameters (distended/contracted or inflamed gallbladder, overhanging liver edge, and cirrhosis). Their scoring system has a sensitivity of 85% and specificity of 97.8% with a maximum score of 44, with a score of 9 predicting a difficult procedure. Their grading systems incorporated many other surgical challenges, including ease or difficulty with umbilical port entry, gall bladder grasping, adhesiolysis, or dissection of Calot’s triangle and duct clipping. However, these are objectively difficult to measure and score. The presence of a cholecystoenteric fistula will invariably indicate severe inflammation and complexity inevitably resulting in conversion. While the absence of fistula in our scoring system may be viewed as a limitation, this phenomenon is rare enough, and if encountered intraoperatively, may warrant a maximal difficulty score.

Gupta et al. [8] in a validation of the scoring system proposed by Randhawa and colleagues [24] allocated a score ranging from 0 (easy) to 15 for the very difficult gallbladder. However, Gupta describes very few operative features- only an ultrasonographically thickened (≥4 mm) GB wall, and an impacted stone in their scoring system. Gallbladder wall thickness is easily measured pre-operatively on US and has been widely used in scoring systems [15,25,26]. Intra-operative measurement of thickness, while technically possible, is not practical in day-to-day surgery and has not been incorporated into our scoring system. Classification of cholecystitis such as the Tokyo Consensus [27] are used to help determine outcomes in studies evaluating treatment modalities in cholecystitis defined as the presence of local inflammation (Murphy sign or right upper quadrant mass, or tenderness) and systemic inflammation (temperature >38°C, elevated C-reactive protein [CRP] levels [>5 mg/L] or an elevated white blood cell count >10 000/μL) and imaging findings (gallstone or biliary debris with a gallbladder wall thickness >4 mm , enlarged gallbladder (long-axis diameter >8 cm and short-axis diameter >4 cm), pericholecystic fluid collection, or linear high density areas in the pericholecystic fat tissue. Severe acute calculous cholecystitis (grade III) is defined as being accompanied by dysfunctions in any one of the following organs or systems: cardiovascular dysfunction with hypotension requiring treatment, neurological dysfunction (decreased level of consciousness), respiratory dysfunction (PaO_2_/FIO_2_ ratio <300), renal dysfunction (oliguria, creatinine > 2.0 mg/dL liver dysfunction (prothrombin time > 3, international normalized ratio > 2) or platelet count <100 000/μL. Moderate acute calculous cholecystitis (grade II) is accompanied by any of the following parameters: white blood cell count greater than 18 000/μL, a palpable tender mass in the right upper abdominal quadrant, duration of complaints for more than 72 hours, or marked local inflammation (gangrenous cholecystitis, pericholecystic abscess, hepatic abscess, biliary peritonitis, or emphysematous cholecystitis). Cases not meeting criteria for severe or moderate acute calculous cholecystitis are classified as mild (grade I). This classification is not surgical based however, and rather broad.

Regimbeau’s [28] open-label, noninferiority, randomized clinical trial utilises these recognised criteria, however, greater emphasis needs to be paid to the actual degree of operative difficulty as this reflects the degree of inflammation and potential for complications. Solomkin [29] rightly emphasizes the importance of evaluating care and outcomes in cholecystitis but we need to go a step further and recognise the importance of documenting the surgical findings to make outcome analysis more meaningful.

The current scoring system is one of the first to outline key operative findings at laparoscopic cholecystectomy. Its validity needs to be tested in future large prospective series before potentially serving as a template for future database and research into patient outcomes.
